# Air and waterborne microbiome of a pharmaceutical plant provide insights on spatiotemporal variations and community resilience after disturbance

**DOI:** 10.1186/s12866-018-1267-8

**Published:** 2018-10-03

**Authors:** Filippo Pacchioni, Alfonso Esposito, Elisabetta Giacobazzi, Clotilde Bettua, Paolo Struffi, Olivier Jousson

**Affiliations:** Centre for Integrative Biology, University of Trento, Trento, Italy

**Keywords:** Built environment, 16S metagenomics, Microbiome resilience

## Abstract

**Background:**

The presence of microrganisms in pharmaceutical production plant environments is typically monitored by cultural methods, however these cannot detect the unculturable fraction of the microbial community. To get more accurate information on the composition of these indoor microbial communities, both water and air microbiome from a pharmaceutical production plant were profiled by 16S amplicon sequencing.

**Results:**

In the water system, we found taxa which typically characterize surface freshwater, groundwater and oligotrophic environments. The airborne microbiome resulted dominated by taxa usually found in outdoor air in combination with human-associated taxa. The alpha- and beta- diversity values showed that the heat-based sanitization process of the water plant affects the composition of the water microbiome by transiently increasing both diversity and evenness. Taxonomic compositional shifts were also detected in response to sanitization, consisting in an increase of Firmicutes and α-Proteobacteria. On the other hand, seasonality seems to be the main driver of bacterial community composition in air of this work environment.

**Conclusions:**

This approach resulted useful to describe the taxonomy of these indoor microbiomes and could be further applied to other built environments, in which the knowledge of the microbiome composition is of relevance. In addition, this study could assist in the design of new guidelines to improve microbiological quality control in indoor work environments.

**Electronic supplementary material:**

The online version of this article (10.1186/s12866-018-1267-8) contains supplementary material, which is available to authorized users.

## Background

Production environments in pharmaceutical companies are kept under strict control to warrant the highest microbiological quality standards. Water and air in the built environment are known to be derived from external environments and thus usually carry a number of environmental microorganisms, either as free-living cells or as cells bound to dust particles [1]. With regard to air samples, spatial and temporal microbial variability are both connected to the type of building and the use of the environments (cooking, sleeping, personal care, etc). The major drivers of indoor air microbiome composition have been identified to be temperature and moisture, while the sources of diversity are from human-associated and outdoor airborne microbiomes [1, 2]. Several studies focused on private houses and working places (reviewed in [2]), with the assumption that the indoor microbiome may have an impact on human health and, consequently, on productivity. To our knowledge, only two previous studies on the indoor microbiome of pharmaceutical companies were carried out so far, focusing on the airborne microbiome alone [3], or on the process of antibiotic production that may lead to the emergence of antibiotic resistance [4].

In the present work, we aimed at developing a novel approach for microbiological quality control of water and air applicable to professional indoor environments, including pharmaceutical companies. We describe the taxonomic profiles of water and air borne microbiome in an italian pharmaceutical plant based on high-troughput sequencing of 16S rRNA amplicons. The microbial alpha- and beta- diversity values were compared in both space and time. In addition, we evaluated the effect on the waterborne microbiome of a heat-based sanitization process of the water plant. Our approach proved to be applicable for the description of these indoor microbiomes, and could be further applied to other built environments where the knowledge of the microbiome composition is of relevance.

## Results

A total of 54 air samples and 136 water samples were collected during a 12-month period (from July 2016 to June 2017, Table 1). After quality filtering, the number of reads ranged 3.851–286.699 (average 76.994 ± 44.781) for water samples, and 1.117–203.107 (average 55.749 ± 59.369) for air samples. To get the best possible estimate of diversity, water and air samples were rarefied to a depth of 29.290 and 2.633 reads, respectively. Those values were chosen to maximize the reliability of the estimates yet keeping more than the 90% of the samples and a balanced design.

**Table 1:** Sampling design outlining the number of samples collected in each production line for water and in each environment for air samples

Sample type	Site	2016		2017	
		Before	After	Before	After
Water	PW19	3	1	3	7
	PW6	3	1	4	7
	PW5	3	1	3	7
	PW14	3	1	3	7
	PW13	3	1	3	7
	PW30	6	1	3	7
	PW20	3	1	3	7
	PW4	3	1	3	6
	PW9	2	0	0	0
	PW12	2	1	0	0
	PW27	2	0	0	0
	PW23	2	0	0	0
	PW2	2	0	0	0
	PW10	2	0	0	0
	PW11	2	1	0	0
	PW22	2	0	0	0
	PW3	2	1	0	0
Air	PP-P	7		7	
	SP-P	3		4	
	M-P	8		8	
	PP-I	2		2	
	SP-I	2		3	
	M-I	4		4	

*PW*, water sampling point, Rooms: *PP*, primary packaging; *SP*, secondary packaging; *M*, mixture. *P*, pharmaceuticals; *I*, food integrators

Samples are grouped per year and (only for water samples) for samples taken before and after the sanitization

**Fig. 1:**
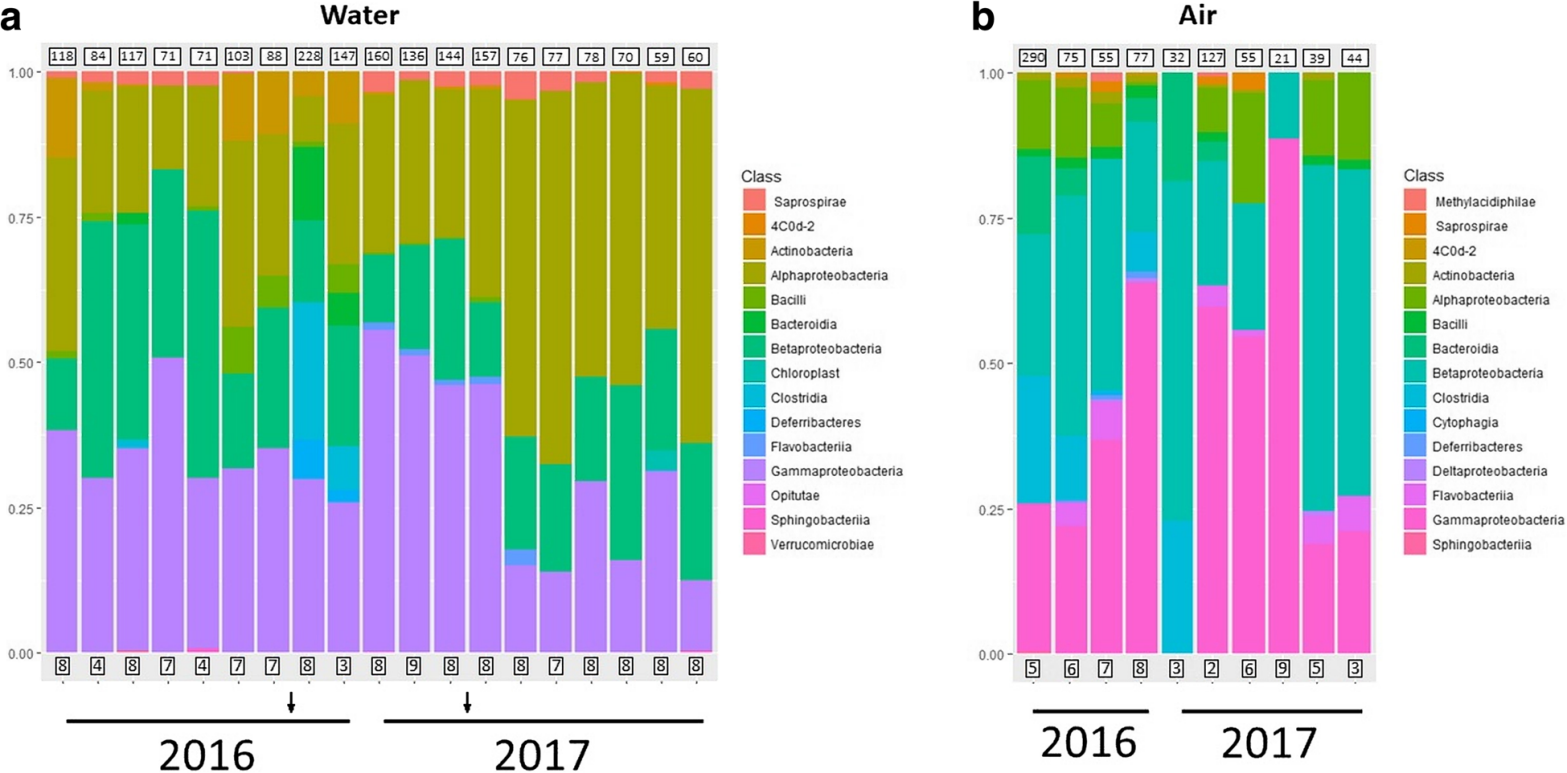
Taxonomic profiles of microbial communities of water (**a**) and air (**b**) samples. Samples collected the same day were grouped into a single bar chart, representing the average value for each taxon. Boxes below histograms represent the number of samples; boxes above the histograms indicate the average number of OTUs obtained for these samples, rounded to the closest entire value. Arrows on the bottom part of the water microbiome chart indicate the time at which sanitization was performed

The microbiome of water samples had higher overall phylogenetic diversity compared to air samples (Fig. 1a and Additional file 1: Figure S1a). The most represented phyla were the Proteobacteria, Firmicutes, Actinobacteria and Bacteroidetes, while other phyla including Cyanobacteria, Verrucomicrobia, Armatimonadetes, and Planctomycetes were also detected at lower abundance. At the class level, α-, β-, γ- and δ-Proteobacteria were the most represented taxa in the Proteobacterial phylum, with a prevalence ranging from 30 to 60% of the samples. Saprospirae (phylum Bacteroidetes) was the most represented non-Proteobacterial class, with a prevalence of 49% of the samples; other Bacteroidetes classes were Flavobacteriia (36%), Sphingobacteriia (28%) and Bacteroidia (11%). Bacilli was the most prevalent class in the Firmicutes with a value of 44% (versus less than 20% of Clostridia).

**Fig. 2:**
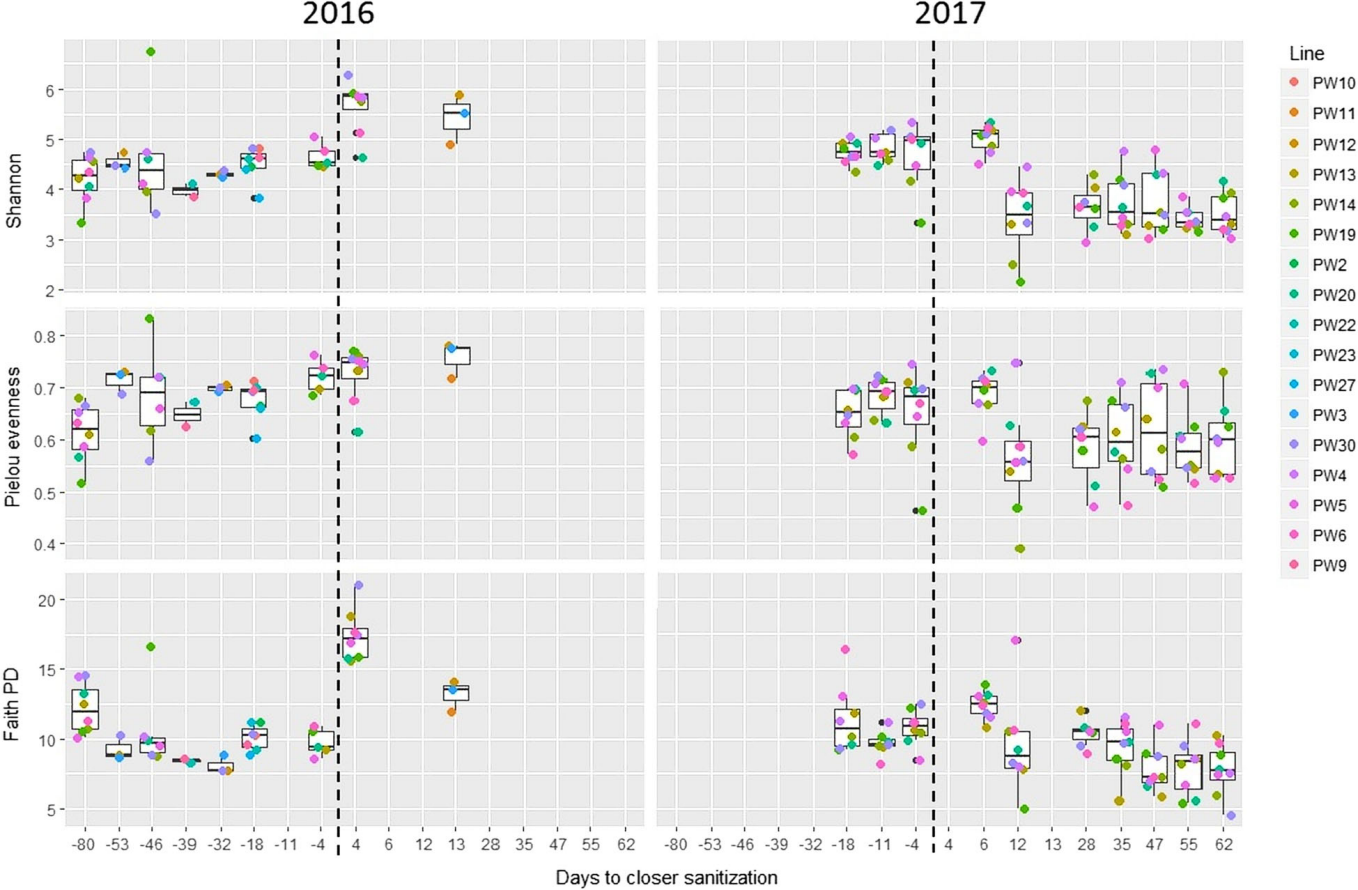
Alpha-diversity values of the water bacterial community (y-axes) as a function of time, expressed as the number of days before (negative values) or after (positive values) sanitization (x-axes). Each dot in the boxplot is a sample and its color represents the production line (PW). The vertical dashed line indicate the time at which sanitization was performed

**Fig. 3:**
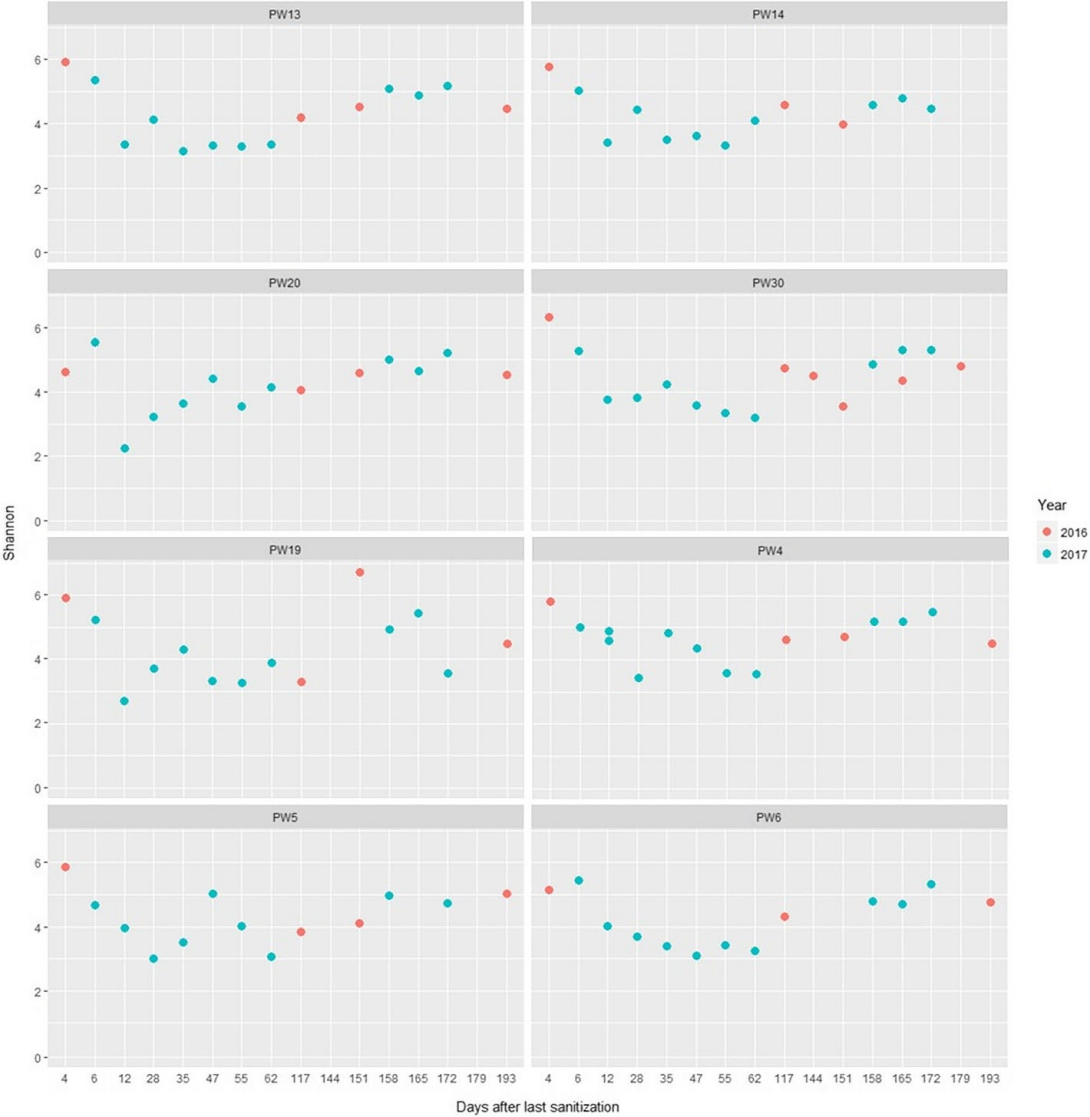
Shannon diversity values (y axis) plotted on the number of days following the last sanitization (x axis) for eight production lines (PW)

All diversity values showed similar trends with respect to the sanitization process: samples collected 4 and 6 days after it showed significantly higher values of all alpha diversity parameters compared to before sanitization, as well as after 10 days onward (Fig. 2). Alpha-diversity values also displayed a weak but significant negative correlation (ranging − 0.28 to − 0.45) with the absolute values of days to the nearest sanitization. This pattern of a transient increase of diversity immediately after sanitization, followed by a drop below pre-sanitization values, was observed in both years of sampling (Fig. 2). Following sanitization, the microbial community is thus composed of less dominant taxa with a better representation of taxa from the low abundance tail, as confirmed from the significantly higher evenness of the samples immediately after sanitization (Fig. 2). To get further insights about such dynamics, we examined the variation of the Shannon diversity value for the eight production lines that were sampled in both years (namely PW4, PW5, PW6, PW13, PW14, PW19, PW20 and PW30). In all cases, the values in the first 6 days were among the highest ones. Such pattern of peaking diversity immediately after sanitization, followed by a drop below the values pre-sanitization for a time span of weeks-to-months was consistently found among the lines and in both years of sampling (Fig. 3).

**Fig. 4:**
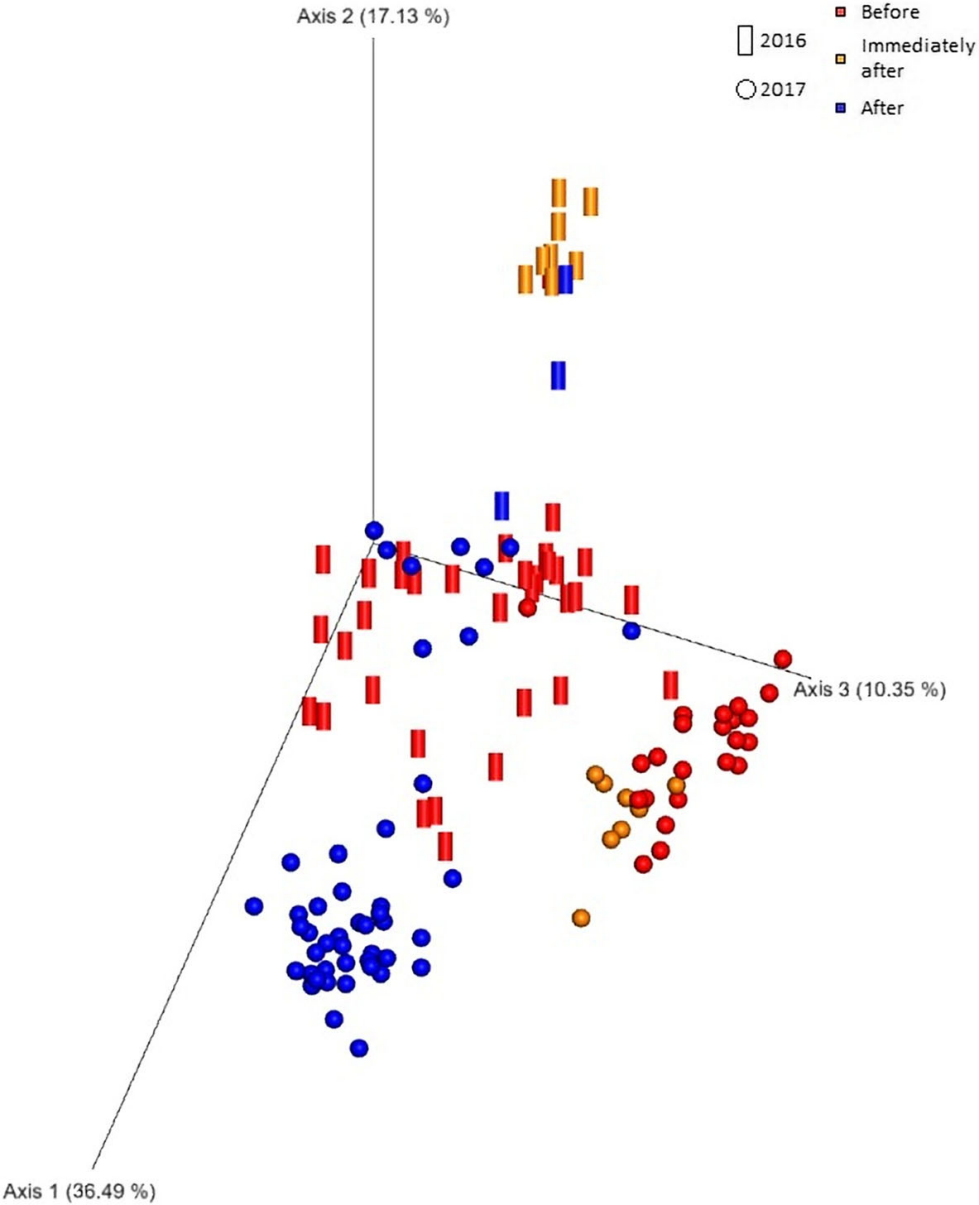
Principal Coordinate Analysis based on the weighted UniFrac distance matrix of water samples. The clustering pattern shows the effects of sanitization in 2016 (cylinders) and 2017 (spheres)

Principal coordinates analysis on the weighted UniFrac distance matrix explained 36.49%, 17.13% and 10.35% of variability on each of the first three axes, respectively (Fig. 4). A cluster of samples, dividing those collected immediately after the sanitization performed in 2016 from all the other ones, is visible on the second axis. The third axis, instead, displays two groups: one constituted by samples collected before and immediately after sanitization and another one with the samples collected more than 10 days after sanitization. The same analysis, performed on the distance matrix obtained from the unweighted UniFrac, produced very similar results, although the variance displayed by the axes was lower (data not shown). The microbiome composition was significantly different among the three samples, as suggested by the PERMANOVA test on the Bray-Curtis distance matrix (Additional file 2: Table S1).

The airborne microbiome, resulted instead dominated by taxa that are usually associated with the outdoor environment such as the order Burkholderiales (β-Proteobacteria, with the remarkable prevalence of 100% for one OTU), and the order Pseudomonadales (γ-Proteobacteria, although this order is also known to be associated with human skin). In addition, two other orders, Enterobacteriales (γ-Proteobacteria) and Lactobacillales (Bacilli), contain taxa of putative human origin (Fig. 1b, Additional file 1: Figure S1b).

An OTU putatively classified as *Acinetobacter lwoffii* was the only taxon significantly more abundant in air samples compared to water samples (Table 2). Within air samples, five taxa were significantly more abundant in samples from 2016 compared to 2017. In water samples, 13 taxa were differentially abundant before and after sanitization, and in seven cases a significant increase was detected immediately after compared to before sanitization (Table 2).

**Table 2:** Differentially abundant taxa between water and air, between air samples in year 2016 and 2017 and between water samples before, immediately after sanitization (< 10 days), and after more than 10 days from sanitization

	Water vs. air(*)	Air 2016 vs. air 2017(**)	Water before vs. immediately after vs. after (***)
Class	4C0D-2 (Melainabacteria) (W)	/	/
Order	/	/	Legionellales (BvI)
Family	Caulobacteraceae (W), Comamonadaceae (W), Pseudomonadaceae (W), Sinobacteraceae (W)	Lachnospiraceae (16), Ruminococcaceae (16)	Sinobacteraceae (AvB, AvI)
Genus	*Sediminibacterium* (W), *Bradyrhizobium* (W), *Methylobacterium* (W), *Sphingomonas* (W), *Alishewanella* (W)	*Blautia* (16)	*Parabacteroidetes* (AvB,AvI), *Oscillospira* (BvA,BvI), *Caulobacter* (IvA,IvB), *Hydrogenophaga* (IvA,IvB), *Methylibium* (AvB,IvB), *Ralstonia* (BvA,BvI), *Bdellovibrio* (AvB, IvB)
Species	*Acinetobacter lwoffii* (A)	*Escherichia coli* (16), *Acinetobacter rhizosphaerae* (16)	*Acinetobacter johnsonii* (IvB, BvA), *Mucispirillum schaedleri* (AvB,BvI), *Bacteroides caccae* (IvB, IvA), *Prevotella copri* (AvB, IvB)

* (W)-more abundant in water, (A)-more abundant in air; ** (16) all samples are more abundant in 2016; *** A, after; B, before; I, immediately after. Ternary comparisons are codified as follows: “time point at which the taxa is more abundant” versus “time point at which the taxa is less abundant”. Example: “BvI” stands for “taxon more abundant before than immediately after sanitization”

## Discussion

The present study consists in an extensive sampling of both water and air across 2 years along the production plant of an Italian pharmaceutical plant. Water microbiome had a higher average number of taxa detected, probably due to the higher number of samples, but also to the effect of the sanitization process that, resulting in a taxonomical shift of the community, led to the detection of a larger number of taxa (Fig. 1a). The water microbiome includes taxa usually found in surface freshwater environments, such as the class Flavobacteriia and the phylum Planctomycetes, and from groundwater and oligotrophic environments, such as Melainabacteria (also known as cyanobacterial class 4C0d-2) and Deferribacteraceae. The latter two classes have peculiar metabolic features: Melainabacteria are non-photosyntetic, obligate fermenters cyanobacteria [5], whereas Deferribacteraceae include species which make energy by anaerobic respiration, using iron as a terminal electron acceptor [6].

The peaks of diversity consistently detected in all lines and at both years of sampling immediately after sanitization clearly show that this process constitutes a disturbance event for the microbial community settled in the water plant. A possible interpretation of this pattern could be that the sanitization selectively affects highly abundant species, allowing fast-growing species from the low-abundance tail of the microbiome to take over for a short time. These typical succession and recovery stages following disturbance have been previously reported for soil bacterial communities [7].

The taxonomic profile of the air microbiome is consistent with prior findings on indoor airborne microbiome, which typically consists in a combination of human-associated and environmental taxa [1, 2]. Surprisingly, the plant’s indoor airborne microbiome does not show marked spatial patterns, as no or little significant differences in neither alpha nor beta diversity were detected among the different rooms. A possible explanation is that the environmental parameters that drive air microbiome composition (mainly moisture and temperature), together with the room usage, are more homogeneous in this setting than in private house rooms. Conversely, significant correlations of both alpha diversity values and beta diversity distance with the sampling date were detected, with a Spearman’s ρ of − 0.64 for Shannon values and 0.37 (after Mantel test) for the weighted UniFrac (in both cases with *p* < 0.01), suggesting that seasonality is the strongest driver of microbial diversity in this indoor environment.

Seven taxa were found at significantly higher abundance immediately after the sanitization (Table 2). At least two possible facts could explain this increase: i) the treatment may have disrupted biofilms formed in peculiar microniches of the plant and killed detached cells, and thus detected DNA might derive from dead cells; ii) resistance stages such as endospores allowed some taxa to survive sanitization. This is most probably the case given the clear increase of the spore-forming class Clostridia that were detected in samples immediately after the sanitization of 2016, and is consistent with previous findings where Clostridia experience a peak of abundance immediately after a thermal stress in soil samples [7].

In a previous study comparing urban wastewater and surface waters before and after disinfection, the authors reported that 3 days after disinfection members of proteobacterian classes, and more specifically, the genera *Pseudomonas*, *Acinetobacter*, and *Rheinheimera*, had a significantly higher abundance compared to controls [8]. We also found a significant increase in an *Acinetobacter* OTU (putatively classified as *A. johnsonii*) immediately after the disturbance, although the abundance of this taxon after more than 10 days was no more significantly higher.

We observed a discrepancy between the taxonomic composition of the water microbiome immediately after sanitization in 2016 and 2017. In 2016, a marked increase of Firmicutes (class Clostridia) was observed; in 2017, main changes in taxonomic composition deals essentially with the class α-Proteobacteria. The sanitization might have had a different effect in 2017 compared to 2016 probably because the bacterial community composition at the beginning of the process was different. This could be due to seasonality of the water microbiome itself; discrepancies in community composition after disturbance have been previously reported [9, 10], and are probably associated with functional redundancy of bacterial communities.

## Conclusions

The airborne microbiome in the pharmaceutical plant seems to be much more influenced by seasonality than location. For what concerns the water microbiome, the heat-based sanitization process results in a short period of increased diversity and abundance of stress-tolerant species followed by a longer period of low diversity, and by a subsequent return to the original diversity values. This study could assist in the design of new guidelines to improve microbiological quality control in indoor work environments. Future studies should extend the description to other components of the microbiome, including viruses and microbial eukaryotes, by means of shotgun metagenomics.

## Methods

### Description of the company

The plant consists of eight production lines: five pharmaceuticals and three food integrators. For each line, there are three physically isolated macro-areas dedicated to granulation, to primary packaging, and to secondary packaging. The water production plant is used primarily to supply purified water to the production process. It consists in a ionic exchange resin group and a double-stage osmosis membrane system. The pre-treatment is performed by a mixed-bed resin ion exchange system, with automatic regeneration by counter-flow washing with a sodium chloride solution. The plant includes two reverse osmosis filtration units installed in series. In each unit, the water first undergoes mechanical filtration in a 5 μm cartridge filter; then a high pressure pump sends the water to the osmosis filtration unit made up of a series of membranes of the “wound spiral polyamide” type. The level of salinity is taken in the first stage from approximately 700 ppm to approximately 5 ppm (TDS); in the second stage, it is reduced to below 1 ppm (TDS). A centralized control system guarantees constant monitoring of the main reference parameters, including conductivity, pH, redox potential, and generates an alarm if values exceed specifications. The circuit loop distributes the water to the production lines. The purified water that circulates in the circuit is maintained at 15 °C by a cooling device. The purified water sequentially flows through each line; there are 17 taps where sampling was performed.

The main units in the air treatment system (UTA) supply filtered and conditioned air to the production areas. Via main ducts and suitable branches, the air reaches the ceiling diffusers in the rooms and is exhausted via wall grilles located at suitable points near the floor. The proportion of recirculated air / reintegrated air is 70/30. The system can maintain a temperature of 22 ± 3 °C and an average relative humidity not exceeding 20%, constantly over 24 h in all seasons of the year. A double stage de-humidification system consisting of i) glycol-cooled tube bundle heat exchangers and ii) rotary Titanium salt unit (Munters), allows to meet these humidity requirements in the main production premises. Filter groups consisting of coarse filter sets (grade G4), pre-filters (grade F9) and HEPA filters (H13) allow to maintain the manufacturing areas clean and to comply with the requirements of the table above. The cleaning of the ducts is guaranteed by the filtration of grade G3 and H10 of pre-filters and filters installed on the suctions, respectively.

Microbiological quality control of air and water of the plant is done in compliance with the European Pharmacopoeia (9th edition) [11].

### Water sample collection

One hundred thirty-six (136) water samples were collected at the taps along the circuit in two periods: the first one from July to October 2016, and the second one from March to June 2017 (Table 1). In the first period, all sampling points were collected but in the second period, only 8 points were considered (PW4, PW5, PW6, PW13, PW14, PW19, PW20, PW30). The water samples were collected in sterilized glass bottles of 1 L and filtered under a biological hood with 0.22 μm polyethersulfone (PES) membranes (Pall corporation).

### Air sample collection

Fifty-two (52) air samples were collected during a period of 4 months, starting from November 2016 to February 2017 (Table 1). Air sampling was performed using an automated air sampler (SAS-super 180; BioScience International, USA). A PES membrane filter was placed onto a TSA agar plate under a biological hood, and a volume of 500 L of air was aspired and conveyed onto the membrane filter for 3 m. Membrane filters were taken from the plate and transferred into a PowerWater Bead Tube for DNA extraction under a biological hood.

### Nucleic acid isolation, amplification of 16S rRNA gene, library preparation and sequencing

DNA extraction was performed under a biological hood. Total DNA from air and water samples was extracted using DNeasy PowerWater kit (QIAGEN S.r.l, Milano, Italy) with minor modifications: at Step 5 of the protocol, the PowerWater® Beat Tube were heated at 65 °C for 10 min; mechanical cell lysis was extended to 10 min for all samples. All other steps were performed following the manufacturer’s instructions. Extracted DNA was stored at − 20 °C.Three 16S rRNA amplicon libraries were produced, one for water samples in 2016, and two for air and water samples in 2017 (one each). The V4 hypervariable region of the 16S rRNA gene was amplified by polymerase chain reaction (PCR) using the 5PRIME HotMasterMix (Quanta BIO), employing 10 μl of MasterMix, 2 μl of extracted DNA, 0.5 μl of each primer at a final concentration of 10 μM, 12 μl of RT-PCR Grade Water (Ambion, Life Technologies) in a total reaction volume of 25 μl of. The target sequence was amplified using 96 different sets of barcoded 806r (GGACTACHVGGGTWTCTAAT) primers and a unique 515f primer (GTGCCAGCMGCCGCGGTAA) [12]. The length of the amplicons was 390 nucleotides. The following thermal cycling conditions were used on a SimpliAmp Thermal Cycler (Applied Biosystems): 3 min 94 °C for initial denaturation; 45 s at 94 °C, 60 s at 50 °C, 90 s at 72 °C for 35 cycles; 10 min at 72 °C for final elongation. Negative controls were included during sampling and main wet-lab steps. 3 PCR blanks, 4 DNA extraction blanks and 4 DNA extractions from unused filters were prepared, for a total of 11 negative control samples. Amplicons concentration, size range and purity were measured using Agilent high sensitivity (HS)DNA kit on the Bioanalyzer 2100 instrument (Agilent Technologies Italia S.p.A, Milano, Italy). From the molarity estimated using Bioanalyzer at 390 bp, each PCR product was diluted and pooled. The final pool was purified using the Agencourt AMPure XP DNA purification kit following manufacturer’s instructions. Amplicons were sent to LaBSSAH-CIBIO NGS facility of the University of Trento for sequencing on an Illumina MiSeq platform with 2 × 300 bp paired-end protocol.

### Bioinformatic analyses

The raw data were analyzed using qiime2 (https://qiime2.org), adapting the standard pipelines described in the “moving pictures” SOP to our dataset. The DADA2 procedure (as implemented in qiime2), performs all pre-processing steps going from quality trimming to OTU-picking, including: correction of amplicon sequencing errors, filtering of phiX reads (a common Illumina carry-over), removal of chimeric sequences and truncation of low quality ends [13]. We set the parameters –p-trim-left 5 and –p-trunc-len 240 for both forward and reverse read. Then, OTUs from the negative control samples were removed from the remaining samples using the ad-hoc plugin developed in qiime2. Representative sequences were aligned using mafft [14], uninformative positions were masked and a phylogenetic tree was built with fasttree [15], using default parameters. The alpha diversity values calculated on rarefied samples were: i) the Shannon index (quantitative, not phylogeny-aware); ii) the Faith’s phylogenetic diversity (qualitative, phylogeny-aware); iii) the Pielou’s evenness, an index estimating the homogeneity of OTUs abundance. The beta diversity was calculated using both weighted and unweighted UniFrac (quantitative and qualitative analyses, respectively, both considering phylogenetic signal) [16], and Bray-Curtis distance (which does not account for phylogenetic signal). Assessment of significant variation of alpha diversity between categories (i.e. air versus water or before versus after sanitization) was determined using the Kruskal-Wallis test. Correlation of diversity values with quantitative measures such as number of days after sanitization (for water) or number of days after monitoring started (for air) was calculated using the Spearman’s ρ. Beta diversity significance (among categories) and correlations (with quantitative values) were calculated with PERMANOVA and Mantel test, respectively. Differential abundances of taxa between water samples collect before, immediately after the sanitization (i.e. in the first 6 days), and more than 10 days after sanitization was inferred using the function ancom [17].

Taxonomic assignment was given to representative sequences using the most updated version of the Greengenes database (v 13.8) [18]. The feature classifier was trained using the qiime2 classify-sklearn plugin on the database; the same plugin also classifies the reads in the real dataset. BIOM tables for water and air were imported in R using the package phyloseq for a graphical representation of diversity values, taxonomic profiles and prevalence of taxa [19, 20].

## Additional files

Additional file 1: **Figure S1.** Prevalence of OTUs classified at the Order (a, for water samples) and Class (b, for air samples) levels. (PDF 502 kb)

Additional file 2: **Table S1.** PERMANOVA test of microbiome data from the samples before, immediately after and after the sanitization. (XLSX 8 kb)

Additional file 3: Metadata file in .tsv format used for bioinformatics analyses with qiime2. (TSV 21 kb)
