# Supplementary material for: Air and waterborne microbiome of a pharmaceutical plant provide insights on spatiotemporal variations and community resilience after disturbance
Source: BMC Microbiol. 2018 Oct 3;18:124. doi: 10.1186/s12866-018-1267-8 (PMC6171249; doi:10.1186/s12866-018-1267-8)
Supplement: Supplementary file 1 — Figure S1. Prevalence of OTUs classified at the Order (a, for water samples) and Class (b, for air samples) levels. (PDF 502 kb) [file 12866_2018_1267_MOESM1_ESM.pdf]

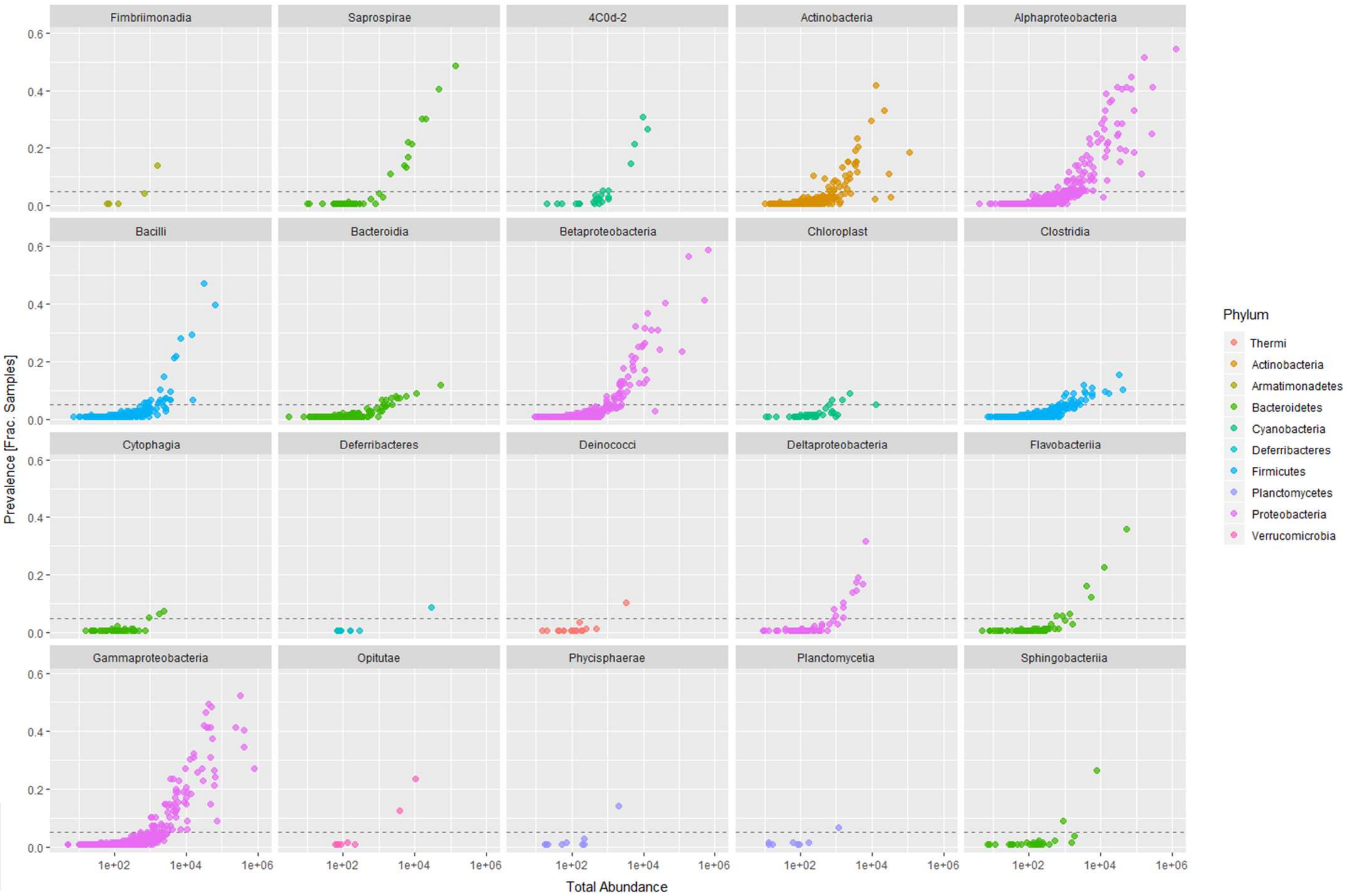

Supplementary Figure 1a - Prevalence of OTUs in water microbiome samples divided by order, expressed as fraction of samples in which an OTU classified within a given order was found. Values on the x-axis represent the absolute number of reads; dots are coloured according to the phylum. Only orders with at least one OTU above the arbitrary threshold of 5% of prevalence are shown.

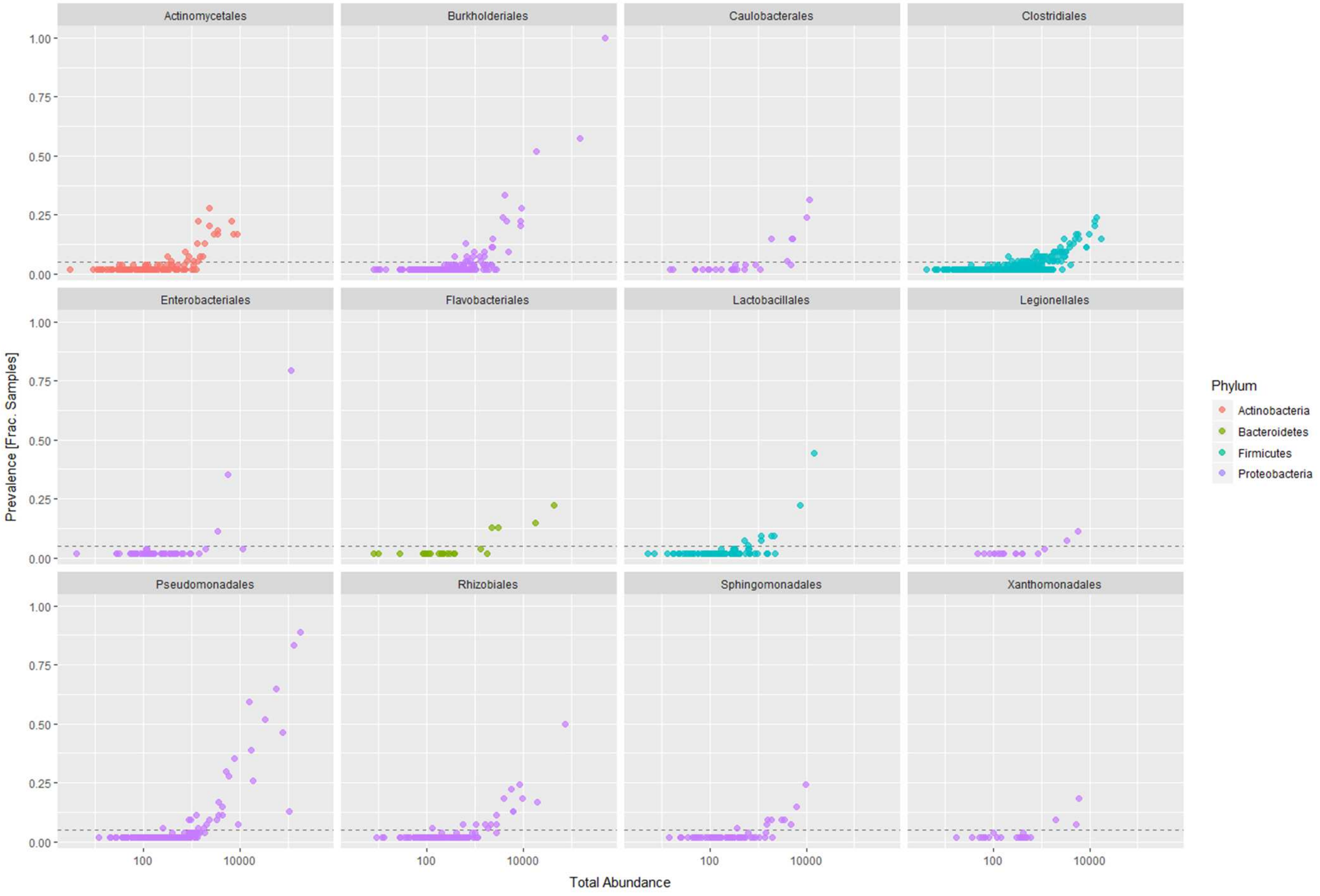

Additional file 1: Figure S1b-Prevalence of OTUs in air microbiome samples divided by class, expressed as fraction of samples in which an OTU classified within a given class was found. Values on the x-axis represent the absolute number of reads; dots are coloured according to the phylum. Only classes with at least one OTU above the arbitrary threshold of 5% of prevalence are shown.
